# Open resection and reconstruction of a Nasoseptal Chondrosarcoma: case report and literature review

**DOI:** 10.1186/s40463-020-00409-6

**Published:** 2020-03-24

**Authors:** Changseok Lee, David Forner, Martin Bullock, Matthew H. Rigby, Martin Corsten, Jonathan R. Trites, S. Mark Taylor

**Affiliations:** 1 Division of Otolaryngology – Head & Neck Surgery, Queen Elizabeth II Health Sciences Center and Dalhousie University, Halifax, Nova Scotia Canada; 2 Department of Pathology, Queen Elizabeth II Health Sciences Center and Dalhousie University, Halifax, Nova Scotia Canada

**Keywords:** Chondrosarcoma, Nasal septum, Head and neck sarcoma, Open rhinoplasty, Tumour, Review

## Abstract

**Background:**

Primary chondrosarcomas of the nasal septum are rare, with a variety of clinical features that evade detection and treatment. While endoscopic surgery has become increasingly accessible, open approaches may be needed to provide adequate visualization for tumour ablation and reconstruction. We report the resection and reconstructive considerations of a septal chondrosarcoma.

**Case presentation:**

A 75-year-old woman presented with a 3-year history of a slow growing, firm mass in the nasal tip causing protrusion and septal fullness. Computed Tomography scan of the paranasal sinuses revealed a well-circumscribed, 2.2 cm mass at the anterior nasal septum extending into the right vestibule. Biopsy of the cartilaginous lesion confirmed the diagnosis of a low-grade chondrosarcoma by histopathology. The tumour was removed using a transcolumellar open rhinoplasty approach with a large septal resection. Primary reconstruction of the surgical defect was performed using an L-shaped strut from the nasal keystone area to the columella. Follow-up examinations demonstrated no evidence of recurrent disease with satisfactory functional and cosmetic outcomes at 1-year.

**Conclusion:**

This report describes a case of nasal septal chondrosarcoma successfully treated with surgical excision using an open rhinoplasty approach. Only 5–10% of chondrosarcomas are located in the head and neck region and arise rarely in the nasal septum in approximately 2–4%. With this mass, an open rhinoplasty approach was required to allow optimal exposure of the margins and to facilitate reconstruction without disruption of normal sinonasal anatomy and function. Although rare, chondrosarcoma of the nasal septum should be considered in the differential diagnosis of nasal masses.

## Background

Chondrosarcoma is a heterogeneous group of tumors that arise from cartilage-producing cells, most frequently involving the pelvis, femurs, and ribs [1]. Head and neck chondrosarcomas are uncommon malignancies affecting the upper aerodigestive tract, and primary chondrosarcomas of the nasal septum are particularly rare. Oncological outcomes for low-grade chondrosarcomas are good, with 5-year survival being up to 89% [2]. Surgical resection is the primary treatment of choice for head and neck chondrosarcoma, with adjuvant radiotherapy and chemotherapy reserved for residual or recurrent disease. The goals of surgical resection are tumor excision with clear specimen margins, and reconstruction with satisfactory functional and aesthetic outcomes.

Recently, endoscopic surgery has emerged as a less invasive option for removal of nasoseptal chondrosarcomas. In the case reported here, the extensive involvement of the cartilage and large resection of the septum necessitated an open rhinoplasty approach to provide adequate exposure for ablation and to facilitate primary reconstruction. The aim of this report is to highlight that while endoscopic techniques are expanding, open surgical approaches may still be required to provide adequate oncologic control and cosmetic considerations. We also provide a summary of the nasoseptal chondrosarcoma literature, including diagnosis, management, and prognosis.

This manuscript was prepared in accordance with the (CARE) guidelines for the reporting of case reports [3].

## Case presentation

A 75-year-old female was referred to the head and neck oncology clinic with a 3-year history of slow-growing mass in the nasal tip that resulted in bulbous tip protrusion and septal fullness. She denied other sinonasal symptoms such as nasal obstruction, pain, discharge, hyposmia, and epistaxis, as well as any associated ocular or neurological changes. Her medical history consisted of hypertension, platelet dysfunction, previous pulmonary embolism, and osteoarthritis; she was a life-time non-smoker. Bimanual examination revealed a firm, nontender mass in the caudal septum palpable on both sides, and no other lesions were identified by flexible endoscopic examination. Her physical examination was otherwise unremarkable, with no palpable cervical lymphadenopathy.

**Fig. 1:**
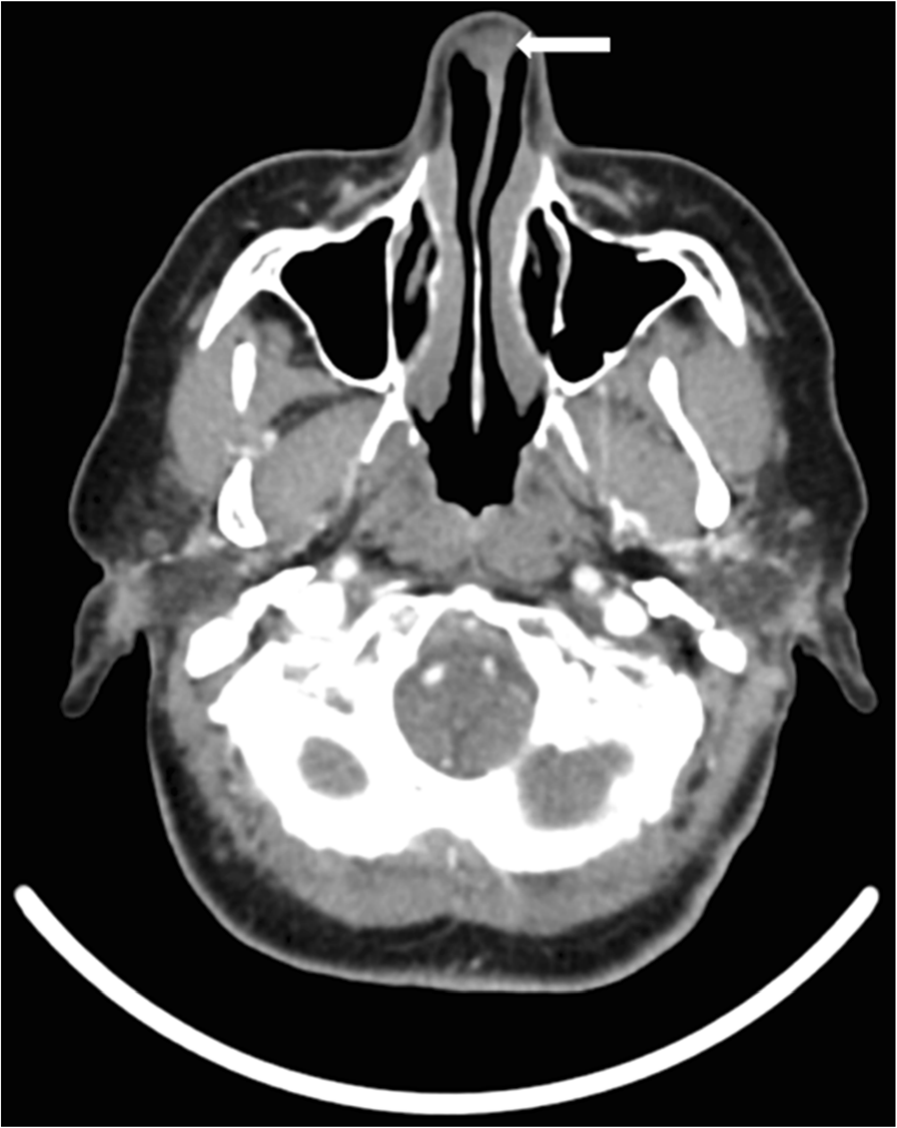
Computed tomography scan showing axial image of 2.2 × 1.5 × 1.0 cm lesion of the caudal nasal septum extending into the right and nasal cavity (arrow). No evidence of bony involvement or destructive growth

Computed tomography (CT) of the paranasal sinuses demonstrated a well-circumscribed lesion arising from the anterior cartilaginous nasal septum. The mass measured 2.2 × 1.5 × 1.0 cm and extended into the right vestibule. There was no evidence of bony erosion (Fig. 1). There was no locoregional or distant metastatic disease identified on imaging studies.

**Fig. 2:**
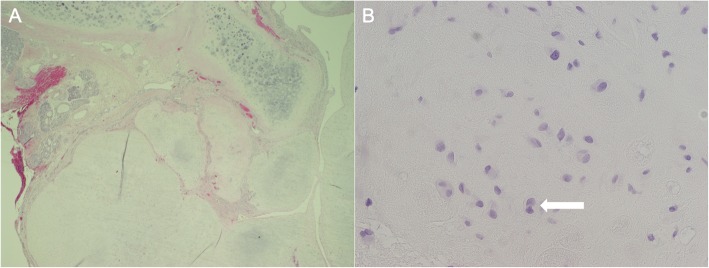
Atypical cartilaginous tumor (grade 1 chondrosarcoma) on hematoxylin and eosin (H&E) staining obtained from the surgical specimen. **a** Low power image (20x), with the lower half of the field occupied by the tumor, abutting nasal mucosa on upper left and native nasal cartilage on upper right. The tumor is lobulated with a pushing border. **b** High power image (400x), showing disorganized chondrocytes with mild nuclear atypia (irregularity), open chromatin and frequent binucleation (arrow)

Biopsy from the left septum revealed a lobulated tumor with borders pushing on nasal mucosa and cartilage. The lobules contained disorganized chondrocytes with nuclear irregularity, open chromatin, and frequent binucleation (Fig. 2). The histology of this cartilaginous neoplasm confirmed the diagnosis of atypical cartilaginous tumor, previously known as grade 1 chondrosarcoma.

**Fig. 3:**
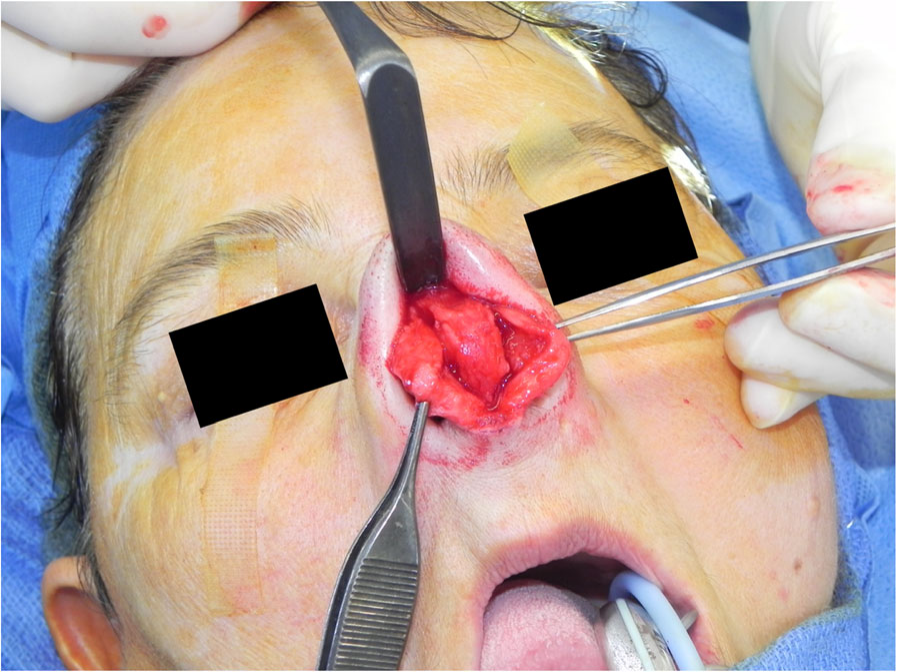
Intra-operative photograph showing tumour arising from the septum

**Fig. 4:**
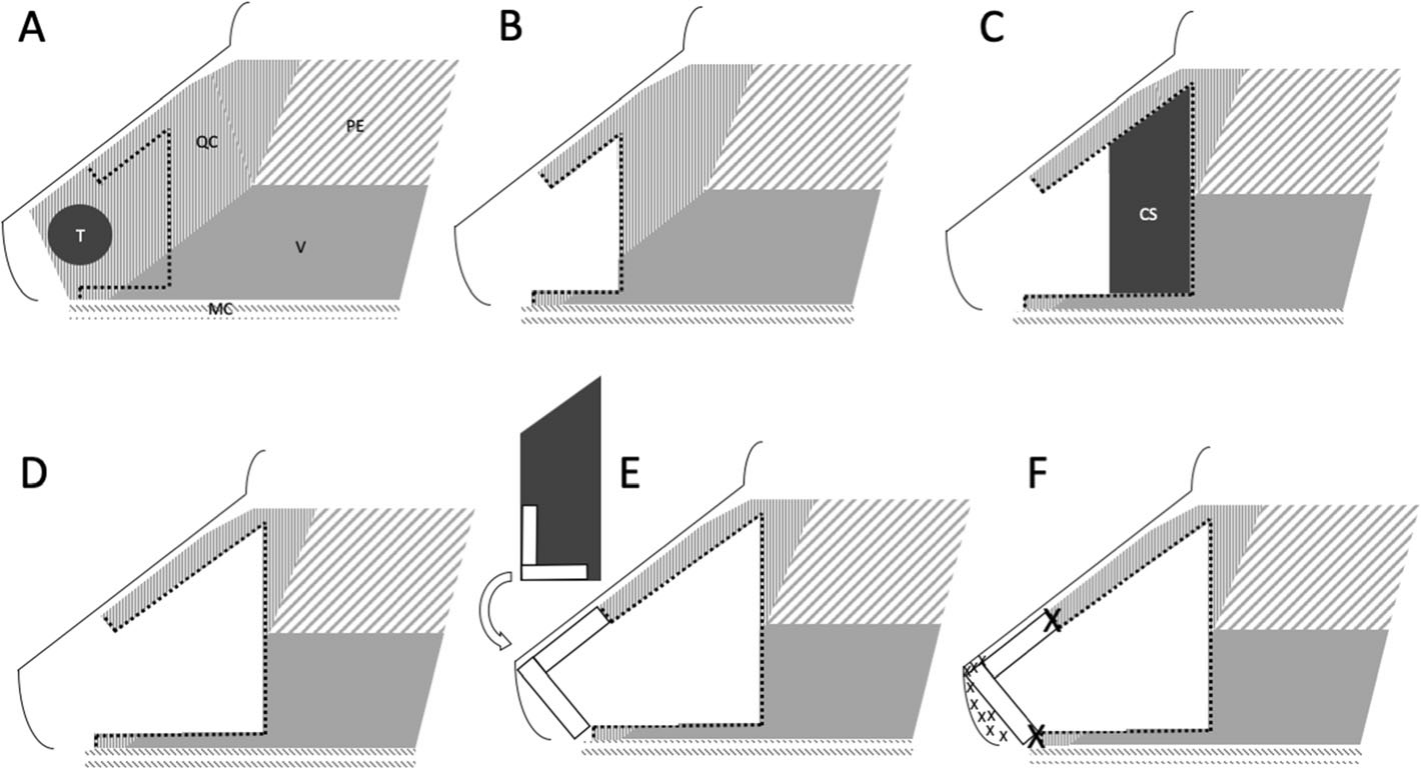
Pictorial diagram of the ablative and reconstructive process. **a** Tumor in situ. **b** Tumor resected, demonstrating defect. **c** Representation of harvested cartilaginous septum for reconstruction. **d** Total defect following oncological and reconstructive resections. **e** Fashioning of L-shaped strut from the cartilaginous septum for reconstruction **f** Completed reconstruction with crushed cartilage graft. (CS: Cartilaginous septum harvested for reconstruction, MC: Maxillary crest, PE: Perpendicular plate of the ethmoid, QC: Quadrangular cartilage, T: Tumor, V: Vomer. Dotted lines represent resection lines, large X represents areas of L-strut fixation, small X represents areas of crushed cartilage augmentation)

The case was presented at the institutional multidisciplinary head and neck tumor board, and open surgical resection was considered the most appropriate management. Extensive tumor involvement of the nasal septum required an open approach to allow adequate exposure and to facilitate reconstruction. Lymphadenectomy of the regional basins was not required. A standard open rhinoplasty approach was employed, with marginal and columellar incisions and division of lower lateral cartilages. Intraoperatively, the dorsal and caudal septum was identified to be involved with chondrosarcoma. The dissection was continued to the nasal dorsum and the upper lateral cartilages were released and preserved on the left. Due to tumor involvement, the upper lateral cartilages on the right side were resected. An inferior cut on the maxillary crest combined with posterior and dorsal incisions of the septum allowed en bloc removal of the tumor (Fig. 3). For reconstruction, mucoperichondrial flaps were raised bilaterally back to the bony septum, and a dorsal strut behind the resection was preserved (Fig. 4a, b). The remainder of the cartilaginous septum was harvested, and an L-shaped strut was fashioned from the keystone area down to the columella. This L-shaped strut was fixed into the remaining columellar strut using 4–0 chromic gut suture (Fig. 4c-e). The opposing end of the L-strut was then attached to the preserved dorsal strut, as well as secured to the residual upper lateral cartilage on the left side, using 4–0 polydioxanone (PDS) suture. The soft tissues of the keystone area were also sutured to the L-shaped strut using PDS suture. Crushed cartilage grafts were generated from left-over harvested cartilaginous septum, applied to the supratip area and columella to camouflage potential nasal dorsum and tip defects (Fig. 4f). Soft tissue closure was then completed in a standard fashion using 4–0 chromic and 5–0 fast absorbing gut sutures, and 6–0 nylon suture. A cartilaginous septal defect remained from the donor area; however, the mucoperichondrium and mucosa allowed satisfactory coverage bilaterally. All tumour margins were histologically negative. There were no post-operative complications, and no adjuvant treatment was required.

**Fig. 5:**
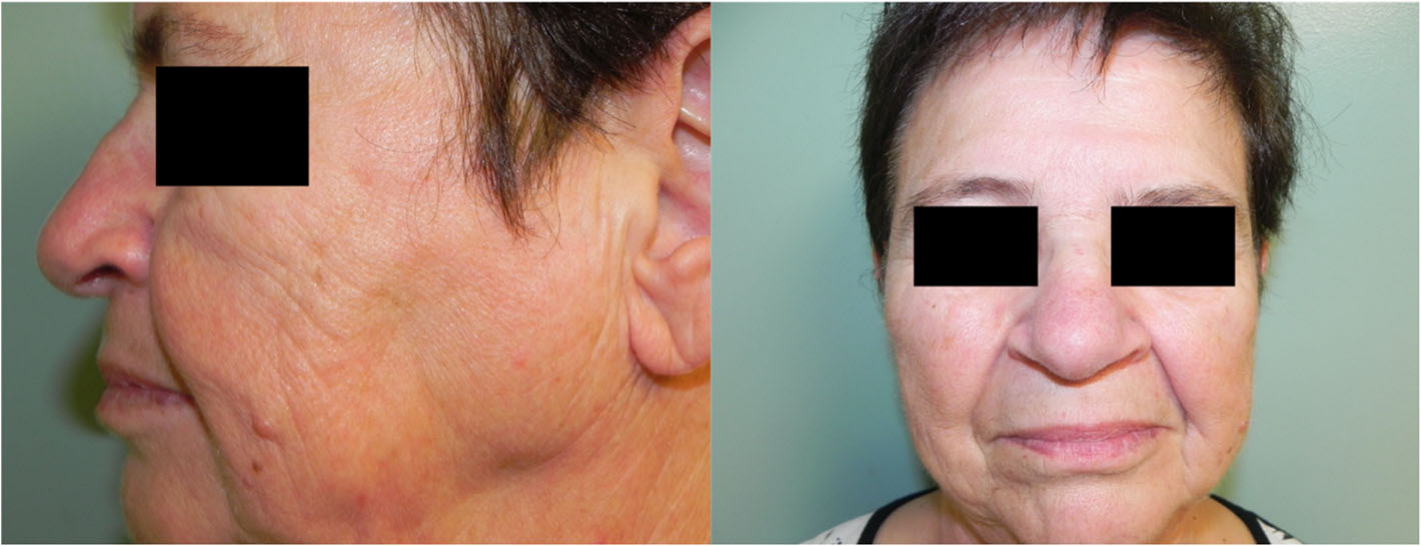
One-year post-operative photographs showing excellent cosmetic results and no evidence of supratip or nasal dorsum defects

On follow-up, the patient remained free of disease at 1 year, with no evidence of residual disease or recurrence. The operative site showed appropriate healing with no septal perforation or nasal cavity obstruction. The cosmetic and functional outcomes were also satisfactory, with well-healed incisions, no saddling of the nasal dorsum, and excellent nasal airflow (Fig. 5). Long-term regular surveillance is ongoing.

## Discussion

Sarcomas in the head and neck are uncommon, representing less than 1% of head and neck cancers [4, 5]. Primary chondrosarcomas of the head and neck represent only 0.1% of tumors in the region. Those originating from the nasal septum are rarer still, captured by fewer than 100 retrospective cases in the existing literature [6, 7]. We present here the case of a large nasoseptal chondrosarcoma requiring open resection through a rhinoplasty approach to facilitate excellent oncological and functional outcomes.

Chondrosarcomas are classified into four main types by their primary site, histology, and prognoses: conventional, clear cell, dedifferentiated, and mesenchymal [6]. Conventional tumors comprise 85% of all chondrosarcomas and can be further subcategorized into primary central, secondary peripheral, and periosteal, depending on their location in bone [1]. Clear cell, dedifferentiated, and mesenchymal chondrosarcomas are rare subtypes and together constitute 10–15% of all chondrosarcomas. These chondrosarcomas are now categorized under malignant chondrogenic tumors [1].

Chondrosarcomas produce cartilage matrix and are histopathologically categorized with three different grades predicting clinical behaviour [1]. Grade I chondrosarcoma—now officially termed atypical cartilaginous tumour [8]—is characterized by the presence of small, dense nuclei and abundant hyaline cartilage matrix, with low metastatic potential [9]. Grade II tumors are intermediate risk, and are characterized by low mitotic rate, intermediate cellularity toward the periphery of tumour lobules, and pale nuclei [7]. In contrast, grade III chondrosarcomas feature high cellularity and mitoses, with large nuclear size and mucomyxoid matrix, progressing to metastatic disease in 70% of patients [7, 10]. Interestingly, chondrosarcoma arising from the head and neck region has been shown to be represented by a smaller proportion of high-grade tumours than those of non-head and neck sites [5]. Histological grading and distinction between benign and malignant chondroid neoplasms remain challenging and are subject to interobserver variability [11]. Although several candidate biomarkers have been proposed to predict differentiative potential, further studies are required to validate their use in tumor grading and prognostics [12, 13].

The average age of sinonasal chondrosarcoma diagnosis is 42.3 years, and there is a slight preponderance toward females (1.27:1). Chondrosarcomas are capable of local invasion and destruction of adjacent structures, and late stages may affect multiple surrounding sites. The majority of tumors grow slowly and are often large at initial detection. The clinical presentation of nasoseptal chondrosarcoma depends on the structures involved, such as the nasal cavity, orbit, paranasal sinuses, Eustachian tubes, or other craniopharyngeal or intracranial compartments [7, 14]. The most frequently reported presenting symptom is nasal obstruction, but patients may also present with a variety of other sinonasal symptoms, such as nasal fullness and chronic discharge, chronic sinusitis, epistaxis, anosmia, facial asymmetry and pain, headaches, and lacrimation. When adjacent structures become involved, conjunctival hyperemia, restricted extraocular movement, diplopia, and exophthalmos may occur [7, 15–17]. Nasoseptal chondrosarcomas in particular often present with symmetrical growth into the nasal cavities with subsequent bilateral symptoms.

Imaging studies are essential to evaluate the osseous and soft-tissue characteristics of the tumor, as well as the extent of its growth to guide appropriate preoperative planning. The most commonly used modalities are CT and MRI. Radiographic findings may demonstrate calcification and cortical bone erosion. Chondrosarcomas typically appears as hypoattenuating matrix with the presence of scattered small, finely speckled, amorphous ring-forming calcifications (also termed “popcorn calcification”), as well as foci of associated erosion of the septum and adjacent bony structures [7, 16]. However, CT may not accurately show the full extent of soft-tissue and intraosseous involvement, and thus MRI is preferable for staging of the tumor.

Surgical resection remains the primary modality of treatment for these tumors. Goals of treatment follow oncological and reconstructive principles and include disease cure, restoration of function, and maintenance of cosmetic outcomes. With surgery, clearance of tumor margins is essential, as the rate of chondrosarcoma recurrence with surgery alone has been reported to increase with positive margins [18]. While the role of routine radiation has not been well defined, radiation has been used preoperatively for large tumors, postoperatively as adjuvant therapy for residual disease, and as salvage therapy for recurrent tumors [15]. Chemotherapy as primary and adjuvant treatment has been used in a small proportion of cases and lacks clear evidence for its benefit in chondrosarcomas [15]. Somatic mutations in the IDH1 and IDH2 genes have also been identified and targeted with specific agents, but the efficacy and role of this targeted molecular therapy is yet to be determined [1, 7].

Radical surgical resection is the mainstay initial treatment of nasoseptal chondrosarcomas. The major cause of mortality is uncontrolled locoregional disease and not metastasis; for low-grade chondrosarcomas, complete excision of the tumor with clear margins may be curative [7]. Wider surgical margins have been shown to decrease the risk of local recurrence. The evidence for margin status in head and neck chondrosarcomas is limited, with highest quality evidence generated from non-head and neck sites. In low-grade chondrosarcoma, margins less than 1 mm are associated with increased risk of recurrence; and in high-grade chondrosarcoma, margins less than 4 mm confer significant risk of recurrence [19]. In two reported cases of head and neck chondrosarcomas, surgical resection with positive margins have been successfully treated with post-operative radiotherapy [14].

With recent advancements of endoscopic endonasal surgery, tumors restricted to the nasal cavity have been shown to be amenable to endoscopic excision with clear margins [7, 17, 20–24]. In select cases of nasoseptal chondrosarcomas, the endoscopic approach has yielded favourable oncological and functional outcomes.

However, characteristics of particular lesions may necessitate an open approach to ensure clear margins and allow for appropriate reconstruction. Due to the location and size of some tumors, the endoscopic approach may not allow adequate visualization for ablation and reconstruction. In the case reported here, the substantial septal resection necessitated primary reconstruction to restore function and offer an acceptable cosmetic outcome. Furthermore, due to the extreme anterior nature of the lesion, surgery performed with traditional endoscopic instruments was expected to be limiting. The open rhinoplasty approach allowed for excellent visualization of the tumor and facilitated the placement of the L-strut for reconstruction. Camouflaging techniques were also employed through the open approach. Both sinonasal function and cosmesis were excellent at follow-up, and the patient remains disease free.

The overall survival of sinonasal chondrosarcoma ranges from 44 to 87% [7]. This wide interval may be due to the rarity of the disease and recent improvements in outcomes as a result of diagnostic and management advancements. With stratification by treatment modalities, 25/35 patients (71.4%) who received surgery and radiation demonstrated disease-free survival of 89.7 months, and 74/116 patients (63.8%) who received surgery alone had no evidence of disease at 36.5 months on average [7]. Of note, head and neck chondrosarcomas have a reduced hazard of disease specific death compared to non-head and neck chondrosarcomas [5]. Local recurrence is relatively common, as seen in 31.1% of patients. Metastasis is rare, but may occur up to 23 years after initial diagnosis and necessitates long-term follow-up with imaging studies and thorough endoscopic examinations [25]. Although rarity of head and neck chondrosarcomas may make management decisions challenging, outcomes have improved substantially over time due to imaging and surgical advancements.

## Conclusion

Although rare, chondrosarcomas of the nasal septum may present with a wide range of sinonasal symptoms and can involve adjacent structures. This case demonstrates a chondrosarcoma with extensive involvement of the septum, requiring an open rhinoplasty approach and primary reconstruction. While endoscopic surgery has recently become a feasible option for lesions limited to the nasal cavity, conventional open surgical approaches should be kept in mind for challenging tumors.

## Data Availability

Not applicable.
